# The Genetic Landscape of Pompe Disease in the Middle East, North Africa, and Turkey (MENAT Region): A Systematic Review of GAA Variants and Genotype–Phenotype Correlation

**DOI:** 10.3390/ijms27209227

**Published:** 2026-10-17

**Authors:** Ahmed Kaid Alantry, Sara Z. Hamdan, Eman A. Kubbara, Mohamad Y. Rezk, Ahmed Mohamedain, Ensherah Hassan Salih Mohamedain, Yousif R. Makki, Ebtihal Kamal, Hamdan Z. Hamdan

**Affiliations:** 1 Department of Basic Health Sciences, College of Applied Medical Sciences, Qassim University, Buraidah 51452, Saudi Arabia; 2 Department of Medicine, Kerry University Hospital, Rathass, V92 NX94 Tralee, Ireland; 3 Clinical Biochemistry Department, Faculty of Medicine, Rabigh Branch, King Abdulaziz University, Rabigh 21911, Saudi Arabia; 4 Department of Physiology, College of Medicine, Qassim University, Buraidah 51452, Saudi Arabia; 5 Department of Biomedical Sciences, College of Medicine, King Faisal University, Al Hofuf 36361, Saudi Arabia; 6 Department of Internal Medicine, King Khalid Hospital, Hail 55421, Saudi Arabia; 7 Department of Basic Medical Sciences, College of Medicine, Prince Sattam Bin Abdulaziz University, Al-Kharj 16273, Saudi Arabia; 8 Department of Pathology, College of Medicine, Qassim University, Buraidah 51452, Saudi Arabia

**Keywords:** children, Pompe disease, glycogen storage disease type II, lysosomal storage disease, genetics, consanguinity

## Abstract

Pompe disease is a rare autosomal recessive multisystem disorder caused by pathogenic variants in the acid alpha-glucosidase (*GAA*) gene, resulting in lysosomal glycogen accumulation and a broad clinical spectrum ranging from rapidly fatal infantile-onset disease (IOPD) to slowly progressive late-onset Pompe disease (LOPD). The genetic epidemiology of Pompe disease across the Middle East, North Africa, and Turkey (MENAT) region remains poorly characterized despite high regional rates of consanguinity. We systematically searched PubMed, ScienceDirect, Google Scholar, and Scopus from inception to July 2026 for studies reporting *GAA* variants in patients with Pompe disease from MENAT countries, following the Preferred Reporting Items for Systematic Reviews and Meta-Analyses (PRISMA) guidelines. We included 24 studies comprising 134 patients with Pompe disease, identifying 61 distinct *GAA* variants. Twelve variants were unique to the MENAT region. Missense variants were the most common variant type, accounting for 50.8% of all distinct variants, followed by frameshift variants 19.7%. Egypt contributed the largest number of reported patients (24/134, 17.9%) followed by Saudi Arabia, Iran, and Iraq (18/134, 13.4%) for each country. The most prevalent variant across the MENAT region is the leaky intronic splice-site variant c.-32-13T>G, reported in 11.2% of all patients and in cases from Turkey, Algeria, Iran and Egypt. Of the 61 distinct variants, 35 were associated with IOPD and 24 with LOPD, with c.1064T>C (p.Leu355Pro) and c.2104C>T (p.Arg702Cys) reported in association with both phenotypes. This systematic review identified country-specific variants in the MENAT region related to Pompe disease that are distinct from other reported variants worldwide. Further studies with larger sample sizes are needed to comprehend the genotype–phenotype correlation for Pompe disease in the MENAT region.

## 1. Introduction

Pompe disease (OMIM #232300), also known as glycogen storage disease type II or acid maltase deficiency, is a rare autosomal recessive inborn error of metabolism caused by biallelic pathogenic variants in the acid alpha-glucosidase (*GAA*) gene, mapped to chromosome 17q25.2–q25.3 [1]. GAA is a lysosomal enzyme that catalyzes the degradation of glycogen to glucose through hydrolysis of α-1,4- and α-1,6-glycosidic linkages [2]. GAA deficiency causes progressive lysosomal glycogen accumulation, particularly in skeletal, cardiac, and respiratory muscles, producing a multisystem disorder with substantial variability in age at onset, disease progression, and clinical severity [3].

Clinically, Pompe disease manifests as a broad continuum rather than a single uniform phenotype. The classic infantile-onset form (IOPD) presents within the first months of life with severe hypotonia, hypertrophic cardiomyopathy, feeding difficulties, and respiratory insufficiency, and is rapidly fatal within the first year of life if left untreated [2,4]. In contrast, late-onset Pompe disease (LOPD) is more heterogeneous, presenting from childhood to late adulthood, and is characterized predominantly by progressive limb-girdle muscle weakness and respiratory muscle involvement, usually without significant cardiac disease [4,5]. This phenotypic variability is largely explained by residual GAA enzymatic activity: patients with virtually absent activity (<1% of normal) typically develop the classic infantile phenotype, whereas residual activity up to 30% of normal is associated with milder, later-onset forms [6].

At the molecular level, this phenotypic spectrum is driven by extensive allelic heterogeneity in the *GAA* gene. More than 600 disease-associated variants have now been cataloged, spanning missense, nonsense, splice-site, and small insertion/deletion changes, as well as larger structural rearrangements, alongside many pseudo deficiency alleles that can complicate diagnostic interpretation [1,7].

The globally reported incidence of Pompe disease is approximately 1 in 40,000 [8]. Incidence varies considerably between countries and populations, ranging from as high as 1 in 14,000 among African American populations to as low as 1 in 600,000 among Portuguese populations, a variation that appears to reflect underlying differences in ethnicity and population genetic structure. Little is known about the incidence or prevalence of Pompe disease specifically within the Middle East, North Africa, and Turkey (MENAT) region. However, a single study from the United Arab Emirates (UAE) reported an incidence of IOPD to be 2.66 per 100,000 [9]. In light of the high rates of consanguineous marriage documented across much of the MENAT region, clinical expression of autosomal recessive disorders including Pompe disease [10,11] is expected to be higher than currently reported [12]. Consensus statements from Gulf-region and Arabian Peninsula expert panels have reinforced concerns about diagnostic delay and under recognition of both IOPD and LOPD across the region, which may lead to underreporting of Pompe disease cases [13,14].

Many countries in the MENAT region have already established national genome projects, including the Saudi Human Genome program [15], the Qatar Genome program [16], the 1000 Arab Genome project in the UAE [17], Iranome [18], the Bahraini Genome Program [19], EgyptRef [20], and the Turkey National Genome and Bioinformatics Project [21]. These initiatives are expected to help clinicians and researchers achieve more accurate insight into and interpretation of genetic findings in regional populations, addressing the current underrepresentation of these nationalities in international genomic databases. A small but growing body of single-country studies has begun to characterize the molecular landscape of Pompe disease locally, including cohorts of Saudi infants with IOPD [22], Egyptian pediatric patients [23,24], and Turkish infants with IOPD [25], many of which report novel or population-specific *GAA* variants that are not well represented in existing international mutation databases. However, these studies remain fragmented, are largely limited to small, single-center cohorts, and have not been synthesized into a comprehensive, region-wide account of the *GAA* variant spectrum across MENAT countries.

A systematic synthesis of the published molecular genetic literature is therefore needed to define the distribution, frequency, and phenotypic correlates of *GAA* variants specific to this region, to identify recurrent or founder alleles, and to highlight gaps in current knowledge that should guide future research and screening strategy. Accordingly, this systematic review aims to comprehensively summarize the published evidence on the spectrum of *GAA* gene variants identified in patients with Pompe disease across the MENAT region.

## 2. Methods

We conducted a detailed systematic search in PubMed, ScienceDirect, Google Scholar, and Scopus from inception to July 2026 to retrieve eligible studies investigating genetic variants associated with Pompe disease in the MENAT region. The Preferred Reporting Items for Systematic Reviews and Meta-Analyses (PRISMA) guidelines were followed as outlined in the PRISMA checklist (Table S1) [26]. The search strategy combined MeSH and non-MeSH terms using the standard Boolean operators [AND], [OR], and [NOT] to maximize search output. The PEO [Participants, Exposure, Outcome] [27] format was followed in the search process for eligible articles to search for articles that investigate the genetic variants related to Pompe disease in the MENAT region as follows:

P [Participants]: patients residing in the MENAT region clinically diagnosed with Pompe disease.

E [Exposure]: Studies reporting pathogenic variants associated with Pompe disease.

O [Outcome]: Pompe disease diagnosis confirmed by lymphocyte acid alpha-1,4-glucosidase (GAA) enzyme levels or genetic analyses.

Table S2 provides the complete list of search terms and the detailed search strategy.

**Figure 1:**
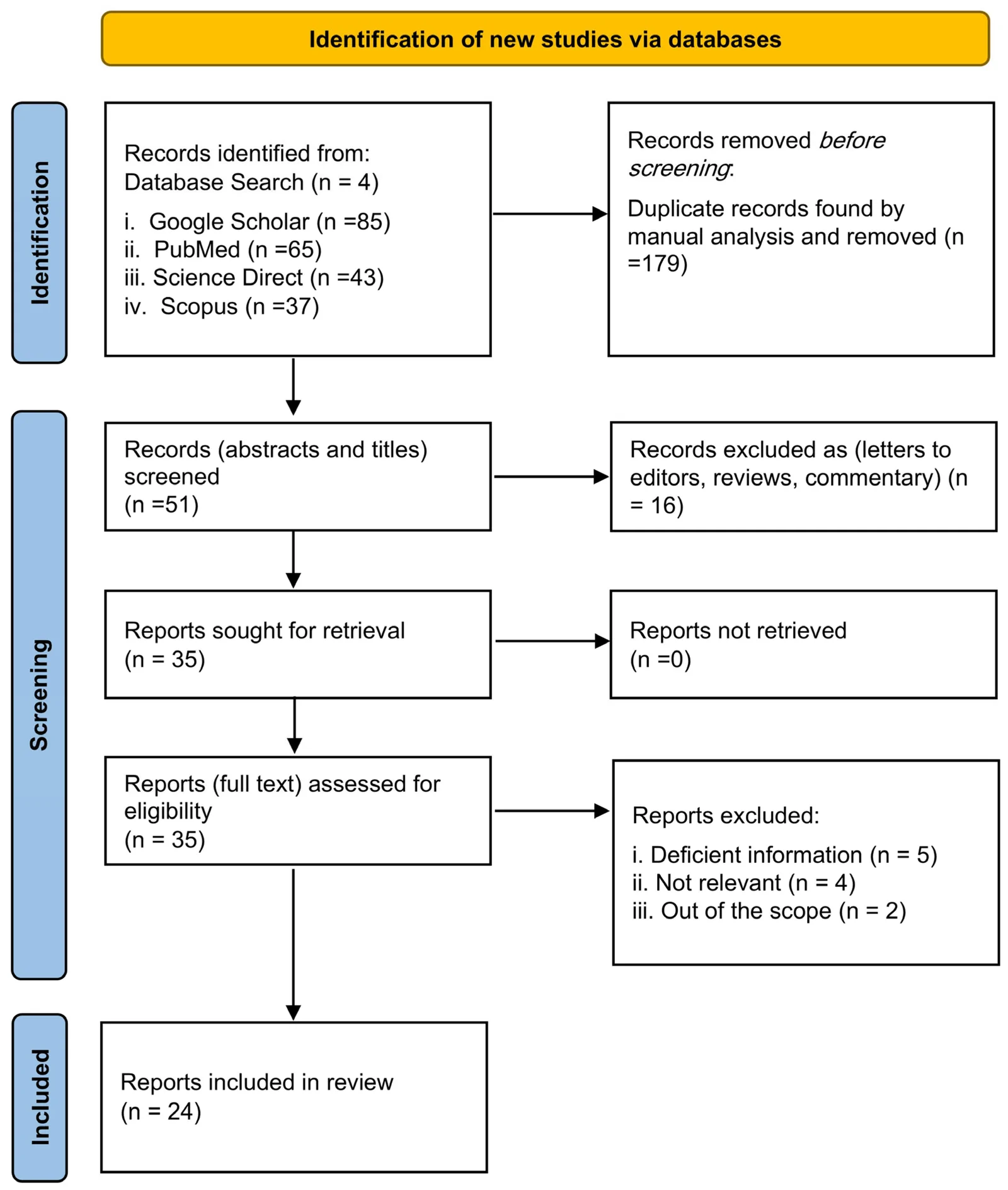
PRISMA flow diagram of the study selection process for the systematic review.

Five investigators (MYR, AKA, SZH, EAK and AM) independently performed the literature search and initial screening of potentially eligible studies. We evaluated titles and abstracts to determine eligibility for full text review and manually screened the reference lists of included articles to identify additional relevant studies. Reviewers resolved disagreements through discussion, with arbitration by a senior reviewer (HZH) when required. We included only articles published in peer-reviewed journals. We excluded duplicate publications, studies in animal models, studies conducted outside the MENAT region, review articles, and non-English articles. The PRISMA flow diagram summarizing the study selection and inclusion process for eligible articles is shown in Figure 1.

### 2.1. Data Extraction

For each included study, four researchers (AKA, MYR, SZH, and AM) independently reviewed the study and extracted the following information: first author’s name; year of publication; country and population studied, number of patients, consanguinity status, Pompe disease subtype (IOPD or LOPD), clinical symptoms, details of the reported *GAA* variant(s), including nucleotide change and amino acid or protein change, and zygosity status.

### 2.2. Definition of the Shared and Unique Variants

To classify variants identified in MENAT-region studies previously reported as variant (shared) or novel (unique), each variant was searched against the following databases: UK Biobank (https://www.ukbiobank.ac.uk/), All of Us research Hub (https://www.researchallofus.org/), FinnGen (https://www.finngen.fi/en), Human Genome Mutation Database (HGMD) (http://www.hgmd.cf.ac.uk/ac/index.php), ClinVar (https://www.ncbi.nlm.nih.gov/clinvar/), gnomAD (https://gnomad.broadinstitute.org/), InterVar (https://wintervar.wglab.org/), and Google Scholar (https://scholar.google.com/), (all aforementioned databases were accessed on 22 July 2026).

A variant was classified as shared if it had been independently reported in any of the aforementioned databases or in studies outside the original MENAT report, and as unique if no independent report was identified. Variants listed in these databases solely on the basis of the same original study included in this review were retained as unique, as such entries do not represent independent observations of the variant.

Variants’ clinical classification as pathogenic, likely pathogenic, conflicting classification, uncertain significance or others were retrieved from ClinVar (https://www.ncbi.nlm.nih.gov/clinvar/) (accessed on 22 July 2026). This clinical classification is based on the ACMG/AMP guidelines [28], which provided a standard framework for variant interpretation.

## 3. Results

The systematic search identified 230 studies, of which 51 remained after duplicate removal (Figure 1). Following title and abstract screening, full texts of 35 studies were retrieved. Of these, 11 were subsequently excluded due to insufficient information, being out of scope, or being irrelevant. Ultimately, 24 articles were selected and included in this systematic review (Table 1) [9,22,23,24,29,30,31,32,33,34,35,36,37,38,39,40,41,42,43,44,45,46,47,48]. Across the included studies, molecular diagnosis confirmed 134 patients with Pompe disease who carried at least one *GAA* variant associated with Pompe disease (Table 1). We identified 61 distinct variants among these patients from 13 MENAT countries (Table 2): 12 (19.6%) were unique to the MENAT region, and 49 (80.3%) were shared with other countries (Table 2).

Most patients with Pompe variants had consanguineous parents (66.4%), and most had a homozygous genotype (70.1%). The IOPD phenotype was present in 69.4% of all patients, while LOPD was present in 30.6%. Out of the 61 variants, 35 variants were related to IOPD and 24 variants were related to the LOPD phenotype, while the variant c.1064T>C (p.Leu355Pro) and c.2104C>T (p.Arg702Cys) were reported in association with both phenotypes.

**Figure 2:**
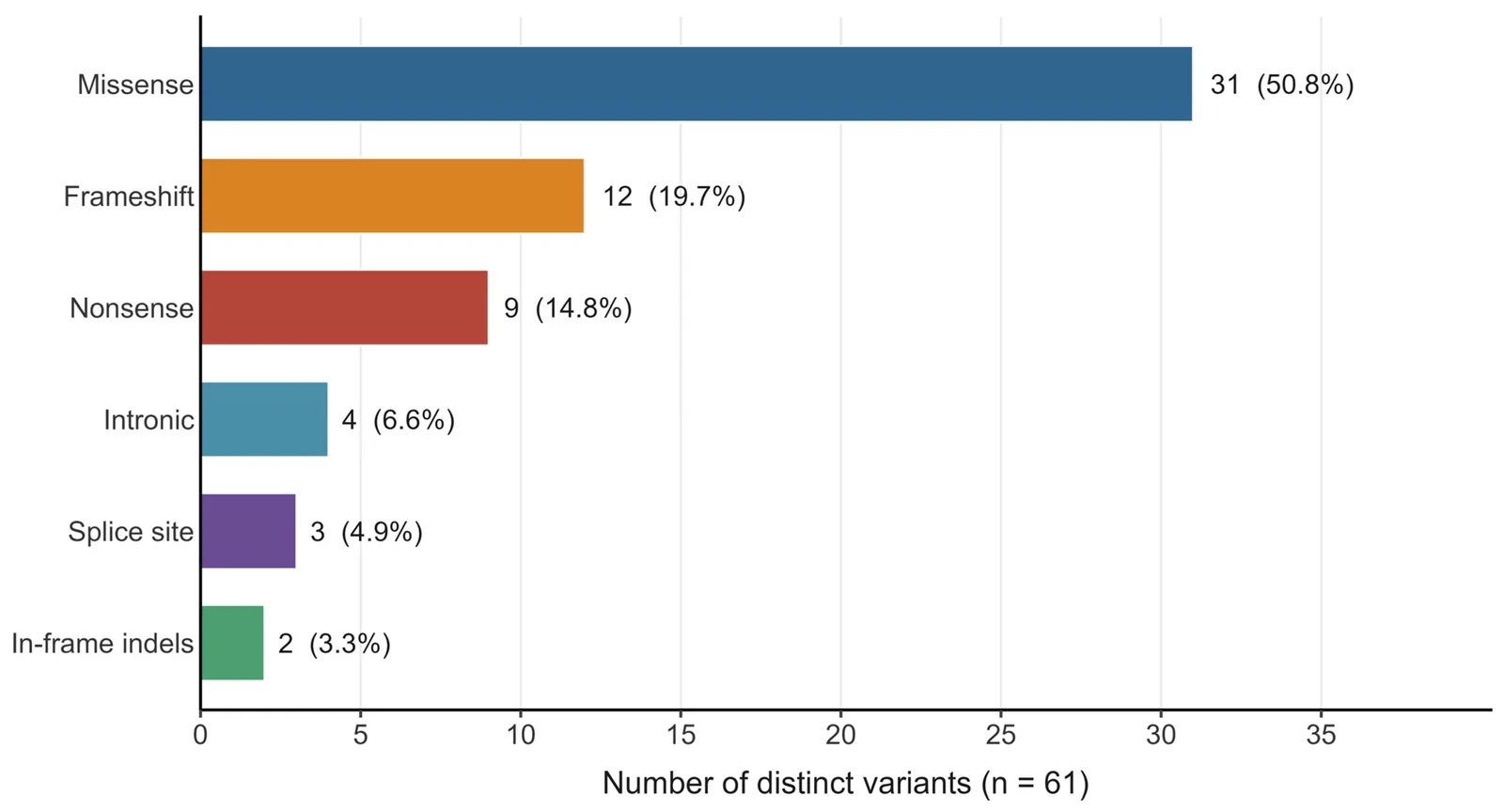
Distribution of distinct *GAA* variants by variant type across the MENAT region.

**Figure 3:**
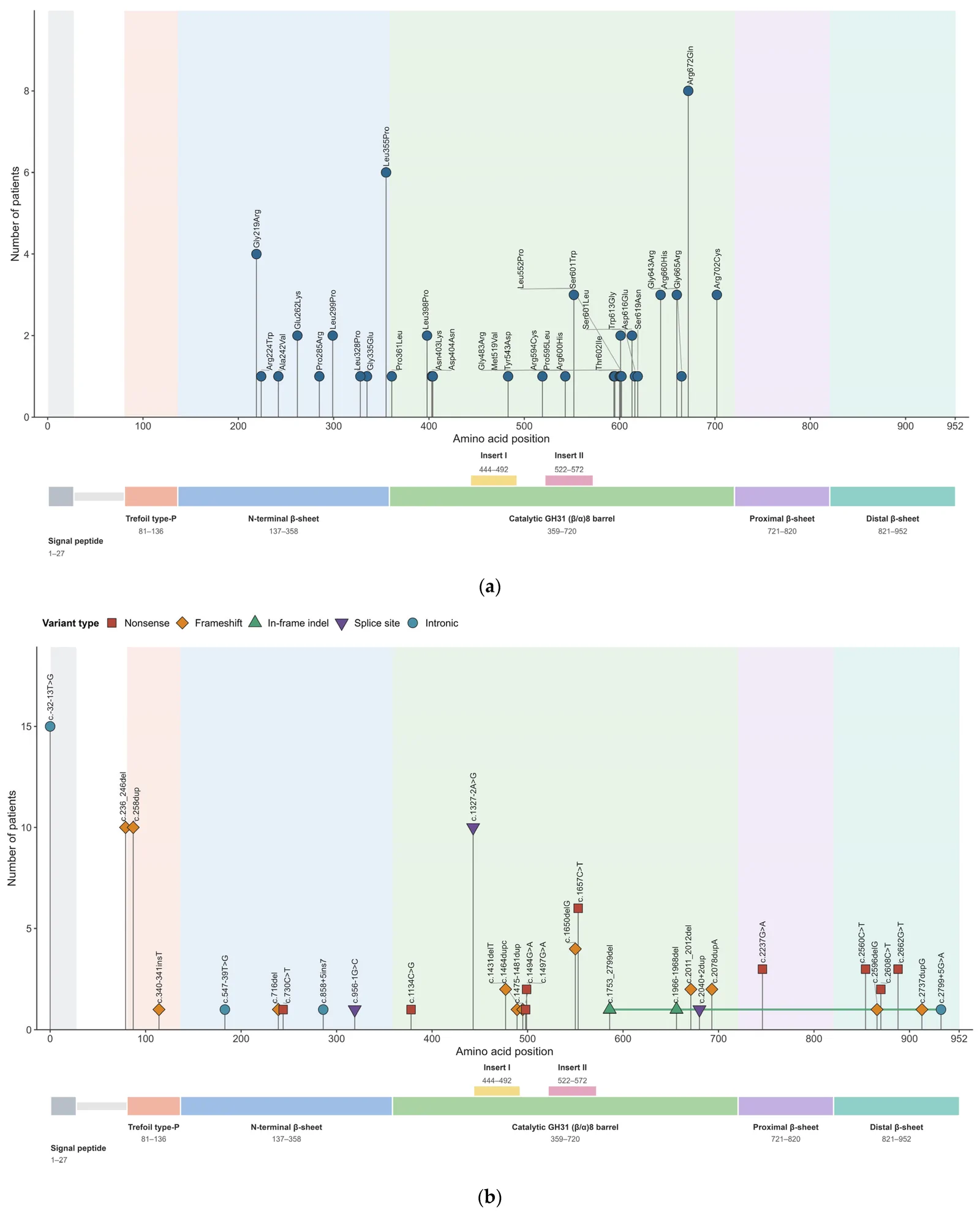
Schematic distribution of Pompe disease-associated *GAA* variants across the GAA protein domains. (**a**) The distribution of missense variants; (**b**) the distribution of other variants that include, nonsense, frameshift, in-frame indel, splice-site and intronic variants.

Among the identified variants, coding variants were the majority: 31 (50.8%) were missense variants, 12 (19.7%) were frameshift variants, 9 (14.8%) were nonsense variants, 1 (1.6%) was an in-frame deletion, and 1 (1.6%) was a large deletion. Noncoding variants included 4 (6.6%) intronic and 3 (4.9%) splice-site variants (Figure 2). Figure 3 presents the schematic localization of the identified missense variants and other variant types 3. A and B, respectively.

By country, the largest number of patients with confirmed *GAA* variants were reported from Egypt (24/134, 17.9%), followed by Saudi Arabia, Iran and Iraq (18/134, 13.4%) for each country, Turkey (14/134, 10.4%), Tunisia (13/134, 9.7%), and the UAE (11/134, 8.2%); see Table 1. Most patients with reported variants had consanguineous parents (66.4%).

**Figure 4:**
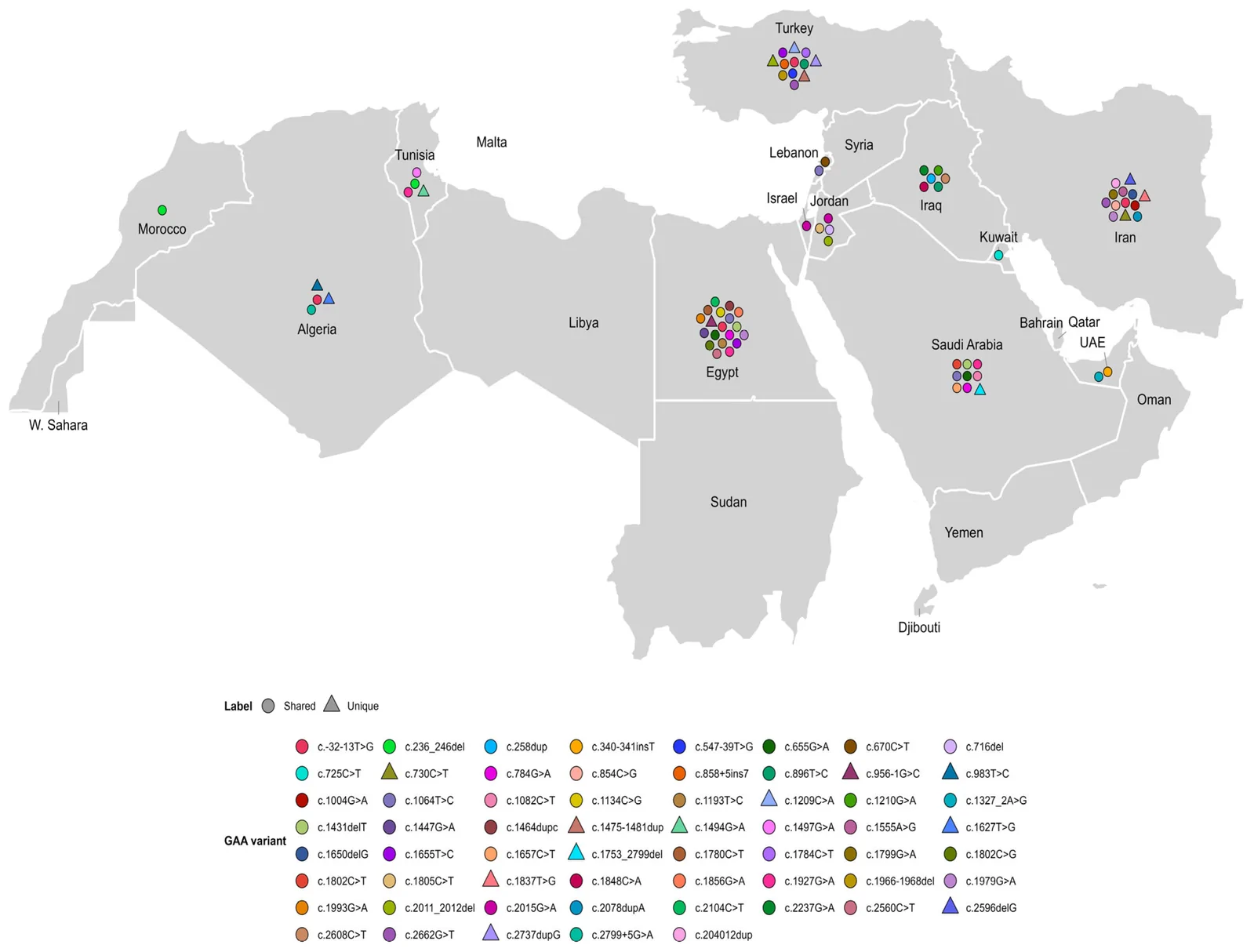
Geographic distribution of the *GAA* variants in the MENAT region.

We identified unique variants in patients from Iran, Turkey, Algeria, Saudi Arabia, Egypt, and Tunisia. The most prevalent variant among MENAT countries is c.-32-13T>G, reported in 4 countries (Algeria, Egypt, Iran, and Turkey), followed by c.1064T>C, reported in Egypt, Lebanon, and Saudi Arabia (see Table 2). Overall, we identified 13 variants shared within the MENAT region and reported in other countries outside the MENAT region. Figure 4 shows the geographic distribution of these variants within the MENAT region, and Table S3 lists them.

## 4. Discussion

This systematic review is the first to summaries the genotype–phenotype correlation of *GAA* variants reported in patients with Pompe disease across the MENAT region. Pompe disease is a monogenic, autosomal recessive disease caused by pathogenic variants in the *GAA* gene, leading to GAA enzyme deficiency and consequent accumulation of glycogen within lysosomes in different tissues, including skeletal muscle, cardiac muscle, and other tissues [1,2]. The GAA enzyme is a large protein of 952 amino acids, organized into six functional domains; namely, an N-terminal region, a trefoil Type-P domain, an N-terminal β-sheet domain, the catalytic GH31 (β/α)_8_ barrel, a proximal β-sheet domain, and a distal β-sheet domain [49].

In this study, we identified 61 distinct variants in the *GAA* gene, of which 19.6 ≈ 20.0% were unique to the MENAT region. The most prevalent variant reported across the MENAT region is c.-32-13T>G, a well-characterized splice-site intronic variant. This variant was reported 15 times among patients from four countries in the MENAT region, namely Algeria [37], Egypt [29], Iran [40], and Turkey [31], either as a homozygous genotype or in compound heterozygosity with other *GAA* variants. Functionally, this variant disrupts splicing of exon 2, producing either complete or partial exon skipping [50]. However, some transcripts escape this aberrant splicing and are processed normally. For this reason, the variant is historically known as the “leaky splicing variant” [51]. Our finding aligns with other reports from Spain [52], the Netherlands [53], Mexico [54], the United States [55], and Russia [56]. Phenotypically, in this study, c.-32-13T>G was associated with the LOPD phenotype, consistent with its capacity to permit partial normal splicing and retain a low but clinically significant level of residual enzyme activity; this phenotype is also recognized in other reports [55].

The second most reported variant related to Pompe disease in the MENAT region is c.236_246del, identified in patients from Tunisia and Morocco. Both Tunisia and Morocco are neighboring Maghrebi countries, and this shared distribution is consistent with the pre-Arab Amazigh (Berber) substrate that underlies much of the modern Maghrebi gene pool [57]. This shared ancestral background offers a plausible rationale for the recurrence of this variant exclusively in these two MENAT countries. Functionally, c.236_246del is an eleven-nucleotide deletion in exon 2 that shifts the reading frame from codon 79 onward (p.Pro79ArgfsTer13); the resulting frameshifted sequence encodes 13 aberrant residues before encountering a premature stop codon [58]. Therefore, transcripts carrying this premature stop codon are expected to be preferentially degraded by nonsense-mediated mRNA decay, and any transcript that escapes this surveillance pathway would be translated into a severely truncated protein that entirely lacks the catalytic domain and retains no enzymatic activity [1]. Clinically, patients homozygous for this variant have consistently developed the classic infantile phenotype, and homozygosity for c.236_246del has been confirmed to be associated with earlier disease onset and a worse prognosis than most other *GAA* variants [59]. A late-onset presentation has also been reported, but exclusively in patients carrying c.236_246del as one allele of a compound heterozygous genotype together with a second, less severe allele [60]. In the present study, all patients homozygous for c.236_246del were classified as having the IOPD phenotype; we also identified one patient with this variant along with a pathogenic missense mitochondrial variant m.12908T>A (p.Leu191Gln) in NADH dehydrogenase subunit 5 [44].

Another notable variant identified in this study is c.1064T>C, a missense variant that changes the p.Leu355Pro in exon 6. This variant was reported in patients from Saudi Arabia [22], Egypt [23,29] and Lebanon [30], and in some cases was associated with IOPD phenotype, while in others was associated with LOPD. Functionally, this variant appears to compromise protein maturation and stability. De Faria and colleagues demonstrate that culture cells transfected with c.1064T>C exhibited severe loss of GAA activity [7], consistent with severe enzymatic deficiency and classic IOPD presentation observed in some of these patients. Of interest, one Lebanese patient carrying the *GAA* c.1064T>C variant also harbored the c.670C>T variant and had a dual diagnosis of IOPD and alkaptonuria, two distinct autosomal recessive inborn errors of metabolism [30]. The coexistence of two rare inherited metabolic disorders in this patient may reflect an increased burden of recessive pathogenic variants within the population, despite the absence of reported parental consanguinity. To the best of our knowledge, this pattern has not been reported elsewhere. Geographically, the c.1064T>C variant spans three different countries in the MENAT sub-region: Saudi Arabia in the Arabian Peninsula, Egypt in North Africa, and Lebanon in the Levant. It is worth mentioning that genome-wide studies of North African populations identified a major pulse of Middle Eastern gene flow into the Maghreb in approximately the 7th century CE, coincident with the Islamic expansion, offering a plausible historical vehicle by which an Arabian-derived allele could have been carried westward into the Tunisian gene pool [61].

We identified twelve variants unique to the MENAT region that have not been reported elsewhere worldwide. Most were reported from Iran, followed by Turkey and Algeria, while Saudi Arabia, Egypt, and Tunisia each reported a single variant. Of these twelve variants, six were reported in association with the IOPD phenotype, while the other six were related to the LOPD phenotype. Iran alone contributed four distinct unique variants, which include a nonsense variant (c.730C>T), a frameshift variant (c.2596delG), a splice-donor variant (c.2040+2dup), and a missense variant (c.1837T>G). Turkey also contributed three distinct novel variants, including a missense variant (c.1209C>A) and two frameshift variants (c.1475_1481dupACCCCAC, and c.2737dupG). Algeria contributed two distinct missense variants (c.1627T>G) and (c.983T>C). The remaining unique variants, including a nonsense variant (c.1494G>A), a splice acceptor variant (c.956-1G>C), and a deletion variant (c.1753_2799del), were identified in Tunisia, Egypt, and Saudi Arabia, respectively. Although the sample sizes of the included studies are limited, the pattern observed here is nonetheless suggestive of a founder effect operating within parts of the MENAT region. This is intensified by high rates of consanguineous and tribal-based marriage, which shapes the current distribution pattern of these pathogenic and unique variants [10].

The recognition of unique variants within the MENAT region may reflect the importance of continued genetic characterization of Pompe disease in this population. Given the expanded neonatal screening program in some MENAT countries, such as Qatar, which recently launched a large-scale genomic newborn screening program for metabolic and inherited diseases aimed at detecting and diagnosing neonates with inherited conditions including Pompe disease [62]. Comprehensive variant recognition is important in this context, as it enables identification of affected cases as early as the neonatal period, allowing earlier intervention and initiation of pharmacological treatment. It is worth mentioning that enzyme replacement therapy (ERT) is the primary pharmacological treatment for Pompe disease. The first-generation ERT, alglucosidase alfa, has significantly improved clinical outcomes, especially survival rates and cardiac function in patients with IOPD [63]. However, limited enzyme uptake by skeletal and respiratory muscles reduces its therapeutic effectiveness. To address this limitation, researchers developed second-generation ERTs such as avalglucosidase alfa. Avalglucosidase alfa contains additional bis-M6P-glycans that enhance binding affinity for the cation-independent M6P receptor (CI-MPR), accordingly improving cellular uptake and lysosomal delivery [64]. This enhancement showed improved tissue uptake and favorable clinical outcomes in patients with LOPD [65] and IOPD especially for patients who transitioned from first generation ERT [66,67,68]. From another perspective, molecular testing can help the patient’s cross-reactive immunological material (CRIM) status. It is widely accepted that variants that yield null enzyme levels are associated with CRIM-negative status. In contrast, variants associated with some protein levels, even if the enzyme is nonfunctional, are associated with CRIM-positive status [69]. Knowing the patient’s CRIM status before initiating ERT is of paramount importance, as it informs the clinical decision to start a preventive immunotolerance protocol, especially in patients with CRIM-negative status [70]. Moreover, CRIM status helps predict the development of anti-GAA antibodies, which are interpreted in terms of disease morbidity and severity [69]. These points help clinicians and genetic counselors inform parental counseling about treatment expectations and immune management. Put together, early molecular diagnosis is important for confirming Pompe disease and deciding whether to initiate ERT promptly, with or without immune modulation.

In this study, we identified 31 distinct missense variants, which account for almost 50% of all variants reported. Three of these are unique to the MENAT region, including c.983T>C (p.Leu328Pro), c.1627T>G (p.Tyr543Asp), both reported in Algeria [37], and c.1837T>G (p.Trp613Gly), reported in Iran [40]. Recent therapeutic advances have introduced pharmacological chaperone therapy (PCT) for Pompe disease [71]. As a standalone approach, PCT can improve mutant enzyme folding and maturation, stabilizing its structure, promoting lysosomal trafficking, and ultimately increasing residual enzymatic activity and improving patient symptoms [72]. PCT may also be administered as an adjunct to ERT to improve its pharmacokinetic characteristics, an approach largely independent of the specific *GAA* variant involved [73]. However, not all patients respond to a standalone PCT approach; this responsiveness depends on the specific variant present, and only missense variants are considered potential candidates for standalone PCT [74]. Evidence showed that around 21% of the missense variants are amenable to PCT [75]. Notably, among the missense variants identified in the MENAT region, only two have been tested for chaperone responsiveness [71]. Those tested include c.1655T>C (p.Leu552Pro), which is found in Turkish patients and was shown to be responsive to PCT, whereas c.1064T>C (p.Leu355Pro), reported from Egypt, Lebanon, and Saudi Arabia, was non-responsive. The remaining variants have not yet been tested for PCT responsiveness.

Another emerging therapeutic approach for Pompe disease is adenine base editing [76]. Experimental studies have demonstrated successful restoration of *GAA* gene expression and activity for three pathogenic nonsense variants, namely; c.2227C>T (p.Q743Ter), c.2560C>T (p.Arg854Ter), and c.2608C>T (p.Arg870Ter) [76]. Of interest, c.2560C>T (p.Arg854Ter) and c.2608C>T (p.Arg870Ter) variants are both represented in the MENAT region and reported from Egypt [29] and Iraq [32], respectively. In the era of personalized medicine, the cornerstone for this approach lies in identifying which variants are amenable to which specific intervention. Therefore, recognizing the pathogenic variants prevalent in the MENAT region may help direct the research efforts toward the targeted variants most likely to benefit patients in this population, in a sort of truly personalized therapeutic approach [50].

Another promising therapeutic approach for Pompe disease is antisense oligonucleotide (AON) therapy [50], which aims to correct aberrant *GAA* pre-mRNA splicing. Accordingly, it is relevant to variants that disrupt normal splicing and result in exon skipping, such as the common c.-32-13T>G variant in this study. Experimental and preclinical studies on fibroblasts from patients with a c.-32-13T>G variant have shown promising results. Demonstrating that splice-modulating AONs can promote correct exon inclusion, increase the production of functional *GAA* transcripts and protein, and restore GAA enzymatic activity to levels above the disease threshold of 20% [50].

This systematic review is the first to summaries variants reported in patients with Pompe disease in the MENAT region and correlate genotype with phenotype. This study provides an important foundation for personalized medicine approaches and for designing neonatal or premarital screening programs to identify carriers of pathogenic *GAA* variants in the MENAT region. However, several limitations warrant consideration. First, the variants were collected from a limited number of studies in the MENAT region, and several countries did not contribute a single published study, so the geographic coverage of this review remains incomplete. Second, the included studies have typically involved small numbers of patients and were largely case series and case reports, and retrospective in nature, raising concerns about how representative these findings are of the broader affected population in each country. Therefore, future studies should include large prospective studies to build a more complete and representative picture of the *GAA* variant spectrum across the MENAT region.

## 5. Conclusions

In conclusion, this systematic review documents the genetic and phenotypic pattern of Pompe disease across the MENAT region, where consanguineous marriages are relatively common. Genotypically, the region is characterized by several country-specific variants not reported elsewhere, associated with the LOPD and IOPD phenotypes equally. The leaky splice variant c.-32-13T>G was the most prevalent variant across the region, while missense variants represented the most common variant type. Identifying shared and country-specific variants provides a regional genetic framework that may guide researchers and clinicians towards targeted molecular diagnostic panels and neonatal and carrier screening strategies. Moreover, defining the variant-specific genotype–phenotype pattern may facilitate interpretation of genetic findings, improve laboratory reporting, enhance genetic counseling and predict the clinical course of Pompe disease. Importantly, many variants in the MENAT region are amenable to the emerging variant-directed therapeutic approaches, including pharmacological chaperone therapy, splice-modulating oligonucleotides, and adenine base-editing approaches. Our study lays the foundation for future functional and clinical trials aimed at transitioning to personalized therapeutic strategies in the MENAT region. Given the limited number of publications from the MENAT region, more studies are needed with larger sample sizes and prospective designs to better cover the population and inform the scientific community about additional variants that contribute to Pompe disease.

## Figures and Tables

**Table 1:** Summary of patients’ demographics and findings captured from 24 studies.

Demographics	Number	%
Variants collected	61	100.0%
Unique variants	12	19.6%
Shared variant	49	80.3%
Patients with variants	134	
**Number of patients with variants by country**		
Algeria	4	3.0%
Egypt	24	17.9%
Iran	18	13.4%
Iraq	18	13.4%
Israel	3	2.2%
Jordan	8	6.0%
Kuwait	1	0.7%
Lebanon	1	0.7%
Morocco	1	0.7%
Tunisia	13	9.7%
Turkey	14	10.4%
Saudi Arabia	18	13.4%
UAE	11	8.2%
**Consanguinity in patients with variants**		
Consanguineous parents	89	66.4%
Non-Consanguineous parents	13	9.7%
Not specified	32	23.9%
**Patients’ zygosity status**		
Homozygous	94	70.1%
Compound heterozygous	22	16.4%
Not available	18	13.4%
**Disease Phenotype**		
LOPD	41	30.6%
IOPD	93	69.4%

Bold text indicates category subheadings.

**Table 2:** Variants captured among Pompe disease patients in the MENAT region.

	Nucleotide Change	Protein Change	Variant Type	Novelty	Phenotype	ClinVar Classification	Number of Patients with the Variant	Country of Origin	Consanguinity
1.	c.1064T>C	p.Leu355Pro	Missense	Shared	IOPD, LOPD	Pathogenic	3, 1, 1	Saudi Arabia [22], Egypt [24,29]	Yes, Yes
2.	c.1082C>T	p.Pro361Leu	Missense	Shared	IOPD	Pathogenic	1	Saudi Arabia [22]	Yes
3.	c.-32-13T>G + c.1209C>A	Intronic + p.Asn403Lys	Intronic + Missense	Shared + Unique	LOPD	Pathogenic + Uncertain significance	1	Turkey [31]	-
4.	c.1210G>A	p.Asp404Asn	Missense	Shared	IOPD	Pathogenic	1	Iraq [32]	Yes
5.	c.1327-2A>G	Splice acceptor	Splice-acceptor	Shared	IOPD	Pathogenic	8, 2	UAE [9,33]	-,Yes
6.	c.1431delT	p.Ile477fs	Frameshift	Shared	IOPD	Pathogenic	1,1	Saudi Arabia [22], Egypt [23]	Yes
7.	c.1464dupC	p.Asp489fs	Frameshift	Shared	LOPD	Pathogenic	1	Egypt [29]	Yes
8.	c.1476_1482dupACCCCAC	p.Ala495Profs	Frameshift	Unique	IOPD	Pathogenic	1	Turkey [34]	Yes
9.	c.1494G>A	p.Trp498Ter	Nonsense	Unique	IOPD	Pathogenic	1	Tunisia [35]	-
10.	c.1497G>A	p.Trp499Ter	Nonsense	Shared	IOPD	Pathogenic	2	Tunisia [35]	Yes
11.	c.1555A>G	p.Met519Val	Missense	Shared	LOPD	Conflicting classification	1	Iran [36]	-
12.	c.1627T>G	p.Tyr543Asp	Missense	Unique	LOPD	Uncertain significance	1	Algeria [37]	Yes
13.	c.1650delG	p.Thr551fs	Frameshift	Shared	LOPD	Pathogenic	2	Iran [38]	-
14.	c.1655T>C	p.Leu552Pro	Missense	Shared	LOPD	Pathogenic	2, 1	Turkey [39], Egypt [24]	-, Yes
15.	c.1657C>T	p.Gln553Ter	Nonsense	Shared	IOPD	Pathogenic	6	Saudi Arabia [22]	Yes
16.	c.1753_2799del	p.Arg586_Lys933del	Deletion	Unique	IOPD	Pathogenic	1	Saudi Arabia [22]	Yes
17.	c.1784C>T	p.Pro595Leu	Missense	Shared	LOPD	Likely pathogenic	1	Turkey [39]	-
18.	c.1802C>T	p.Ser601Leu	Missense	Shared	IOPD	Pathogenic	1	Saudi Arabia [22]	Yes
19.	c.1837T>G	p.Trp613Gly	Missense	Unique	LOPD	Likely pathogenic	2	Iran [40]	Yes
20.	c.1848C>A	p.Asp616Glu	Missense	Shared	IOPD	Conflicting classification	1	Iraq [32]	-
21.	c.1927G>A	p.Gly643Arg	Missense	Shared	IOPD	Pathogenic	1, 1, 1	Saudi Arabia [22], Tunisia [35], Egypt [24]	Yes, -, -
22.	c.1962_1964delGAG	p.Glu656del	Deletion	Shared	IOPD	Pathogenic	1	Turkey [34]	Yes
23.	c.1979G>A	p.Arg660His	Missense	Shared	LOPD	Pathogenic	2, 1	Iran [40], Egypt [24]	Yes, yes
24.	c.1993G>A	p.Gly665Arg	Missense	Shared	LOPD	Uncertain significance	1	Egypt [29]	Yes
25.	c.2011_2012delAT	p.Met671fs	Frameshift	Shared	IOPD	Pathogenic	1, 1	Turkey [34], Jordan [47]	-, Yes
26.	c.2015G>A	p.Arg672Gln	Missense	Shared	IOPD	Likely pathogenic	3, 3, 2	Israel [41], Jordan [42,47]	Yes, yes, yes
27.	c.2040+2dup	Splice donor	Splice donor	Unique	LOPD	Uncertain significance	1	Iran [40]	Yes
28.	c.2104C>T	p.Arg702Cys	Missense	Shared	LOPD, IOPD	Pathogenic	1,2	Egypt [24,29]	Yes, Yes
29.	c.2237G>A	p.Trp746Ter	Nonsense	Shared	IOPD	Pathogenic	3	Iraq [32]	Yes
30.	c.236_246del	p.Pro79fs	Frameshift	Shared	IOPD	Pathogenic	8, 1	Tunisia [35,44], Morocco [43]	Yes, yes, yes
31.	c.236_246del + m.12908T>A ^‡^	p.Pro79fs + Mitochondrial missense	Frameshift + mitochondrial missense	Shared	IOPD	Pathogenic + pathogenic	1	Tunisia [44]	Yes
32.	c.2560C>T	p.Arg854Ter	Nonsense	Shared	LOPD	Pathogenic	3	Egypt [29]	Yes
33.	c.258dup	p.Asn87fs	Frameshift	Shared	IOPD	Pathogenic	10	Iraq [32]	Yes
34.	c.2608C>T	p.Arg870Ter	Nonsense	Shared	IOPD	Pathogenic	2	Iraq [32]	Yes
35.	c.2799+5G>A	Intronic variant	Intronic	Shared	IOPD	Uncertain significance	1	Algeria [37]	Yes
36.	c.-32-13T>G	Intronic variant	Intronic	Shared	LOPD	Pathogenic	1, 1	Turkey [31], Algeria [37]	-, Yes,-,Yes
37.	c.-32-13T>G+c.2662G>T	Intronic variant + p.Glu888Ter	Intronic variant + Nonsense	Shared + shared	LOPD	Pathogenic + pathogenic	2, 1	Turkey [31], Iran [40]	-,-
38.	c.-32-13T>G+c.1856G>A	Intronic variant + p.Ser619Asn	Intronic variant + Missense	Shared + shared	LOPD	Pathogenic + pathogenic	1	Egypt [29]	-
39.	c.-32-13T>G+c.1004G>A	Intronic variant + p.Gly335Glu	Intronic variant + Missense	Shared + shared	LOPD	Pathogenic + Likely pathogenic	1	Iran [40]	-
40.	c.-32-13T>G+c.1650delG	Intronic variant + p.Thr551fs	Intronic variant +Frameshift	Shared + shared	LOPD	Pathogenic + pathogenic	2	Iran [40]	-
41.	c.-32-13T>G+c.1799G>A	Intronic variant + p.Arg600His	Intronic variant + Missense	Shared + shared	LOPD	Pathogenic + pathogenic	1	Iran [40]	-
42.	c.-32-13T>G + c.2737dupG	Intronic variant + Frameshift	Intronic variant + Frameshift	Shared + Unique	LOPD	Pathogenic + pathogenic	1	Turkey [31]	-
43.	c.-32-13T>G + c.2078dupA	Intronic variant + p.Ala694fs	Intronic variant + Frameshift	Shared + shared	LOPD	Pathogenic + pathogenic	2	Iran [40]	Yes
44.	c.-32-13T>G + c.2596delG	Intronic variant + p.Glu866fs	Intronic variant + Frameshift	Shared + Unique	LOPD	Pathogenic + pathogenic	1	Iran [40]	-
45.	c.340_341insT	p.Lys114fs	Frameshift	Shared	IOPD	pathogenic	1	UAE [33]	-
46.	c.858+5ins7 + c.547-39T>G	Intronic variant + Intronic variant	Intronic variant + Intronic variant	Shared + shared	LOPD	Unknown + Benign	2	Turkey [31]	-
47.	c.655G>A	p.Gly219Arg	Missense	Shared	IOPD	Pathogenic	3, 1	Saudi Arabia [22], Egypt [24]	Yes, yes
48.	c.1064T>C + c.670C>T	p.Leu355Pro+ p.Arg224Trp	Missense +Missense	Shared + shared	IOPD	Pathogenic + pathogenic	1	Lebanon [30]	-
49.	c.725C>T	p.Ala242Val	Missense	Shared	LOPD	Uncertain significance	1	Kuwait [48]	-
50.	c.730C>T	p.Gln244Ter	Nonsense	Unique	IOPD	Pathogenic	1	Iran [46]	-
51.	c.1802C>T+ c.784G>A	p.Ser601Leu+ p.Glu262Lys	Missense + Missense	Shared + shared	IOPD	Pathogenic + pathogenic	1	Saudi Arabia [22]	-
52.	c.854C>G	p.Pro285Arg	Missense	Shared	LOPD	Likely pathogenic	1	Iran [40]	Yes
53.	c.896T>C	p.Leu299Pro	Missense	Shared	IOPD	pathogenic	1, 1	Turkey [45], Iraq [32]	Yes, -
54.	c.983T>C	p.Leu328Pro	Missense	Unique	IOPD	Uncertain significance	1	Algeria [37]	Yes
55.	c.716del	p.Leu239Argfs	Frameshift	Shared	IOPD	pathogenic	1	Jordan [47]	Yes
56.	c.1805C>T	p.Thr602Ile	Missense	Shared	IOPD	Likely pathogenic	1	Jordan [47]	Yes
57.	c.1193T>C	p.Leu398Pro	Missense	Shared	IOPD	pathogenic	2	Egypt [23]	Yes
58.	c.1134C>G	p.Tyr378Ter	Nonsense	Shared	IOPD	Pathogenic	1	Egypt [23]	Yes
59.	c.784G>A	p.Glu262Lys	Missense	Shared	IOPD	Pathogenic	1	Egypt [24]	Yes
60.	c.1802C>G	p.Ser601Trp	Missense	Shared	IOPD	Likely pathogenic	1	Egypt [24]	Yes
61.	c.956-1G>C	Splice acceptor	Splice acceptor	Unique	IOPD	Pathogenic	1	Egypt [24]	Yes
62.	c.1780C>T	p.Arg594Cys	Missense	Shared	IOPD	Uncertain significance	1	Egypt [24]	Yes
63.	c.1447G>A	p.Gly483Arg	Missense	Shared	IOPD	Pathogenic	1	Egypt [24]	Yes

^‡^ m.12908T>A is a concurrent mitochondrial variant in NADH dehydrogenase subunit 5 (ND5) variant.

## Data Availability

The original contributions presented in this study are included in the article/Supplementary Material. Further inquiries can be directed to the corresponding author.

## Supplementary Materials

The following supporting information can be downloaded at https://www.mdpi.com/article/10.3390/ijms27209227/s1.
