# Supplementary material for: The Genetic Landscape of Pompe Disease in the Middle East, North Africa, and Turkey (MENAT Region): A Systematic Review of GAA Variants and Genotype–Phenotype Correlation
Source: Int J Mol Sci. 2026 Oct 17;27(20):9227. doi: 10.3390/ijms27209227 (PMC13705103; doi:10.3390/ijms27209227)
Supplement: Supplementary file 1 [file ijms-27-09227-s001.zip › Table S2.pdf]

Table S2: Search strategies for variants related to Pompe disease in the MENAT region

Database	Search Strategy
**PubMed**	("Glycogen Storage Disease Type II"[Mesh] OR Pompe[tiab] OR "glycogen storage disease type II"[tiab] OR "glycogen storage disease type 2"[tiab] OR "glycogen storage disease II"[tiab] OR "glycogenosis type II"[tiab] OR "GSD II"[tiab] OR GSD2[tiab] OR "acid maltase deficiency"[tiab] OR "acid alpha-glucosidase deficiency"[tiab] OR "GAA deficiency"[tiab])<br>**AND**<br>(GAA[tiab] OR gene[tiab] OR genes[tiab] OR genetic*[tiab] OR genotyp*[tiab] OR mutation*[tiab] OR variant*[tiab] OR polymorph*[tiab] OR sequencing[tiab] OR molecular[tiab] OR allele*[tiab])<br>**AND**<br>(Algeria*[tiab] OR Bahrain*[tiab] OR Egypt*[tiab] OR Iran*[tiab] OR Iraq*[tiab] OR Israel*[tiab] OR Jordan*[tiab] OR Kuwait*[tiab] OR Lebanon[tiab] OR Lebanese[tiab] OR Libya*[tiab] OR Morocco[tiab] OR Moroccan*[tiab] OR Oman[tiab] OR Omani[tiab] OR Qatar*[tiab] OR Saudi*[tiab] OR Sudan*[tiab] OR Syria*[tiab] OR Tunis*[tiab] OR Turkey[tiab] OR Türkiye[tiab] OR Turkish[tiab] OR "United Arab Emirates"[tiab] OR Emirat*[tiab] OR UAE[tiab] OR Yemen*[tiab] OR MENAT[tiab] OR MENA[tiab] OR "Middle East"[tiab] OR "Middle Eastern"[tiab] OR "North Africa"[tiab] OR "North African"[tiab])
**Scopus**	TITLE-ABS-KEY((Pompe OR "glycogen storage disease type II" OR "glycogen storage disease type 2" OR "glycogen storage disease II" OR "glycogenosis type II" OR "GSD II" OR GSD2 OR "acid maltase deficiency" OR "acid alpha-glucosidase deficiency" OR "GAA deficiency")<br>**AND**<br>TITLE-ABS-KEY(GAA OR gene OR genes OR genetic* OR genotyp* OR mutation* OR variant* OR polymorph* OR sequencing OR molecular OR allele*)<br>**AND**<br>TITLE-ABS-KEY(Algeria* OR Bahrain* OR Egypt* OR Iran* OR Iraq* OR Israel* OR Jordan* OR Kuwait* OR Lebanon OR Lebanese OR Libya* OR Morocco OR Moroccan* OR Oman OR Omani OR Qatar* OR Saudi* OR Sudan* OR Syria* OR Tunis* OR Turkey OR Türkiye OR Turkish OR "United Arab Emirates" OR Emirat* OR UAE OR Yemen* OR MENAT OR MENA OR "Middle East" OR "Middle Eastern" OR "North Africa" OR "North African"))
**Science Direct**	Pompe OR "glycogen storage disease type II" OR "glycogen storage disease type 2" OR "glycogen storage disease II" OR "acid maltase deficiency" OR "acid alpha-glucosidase deficiency" OR "GAA deficiency" OR "glycogenosis type II"

**Google scholar**	allintitle: POMPE DISEASE OR Glycogen storage disease type II OR Glycogen storage disease type 2 OR GSD II) AND (Gene OR Genetic OR Mutation OR Variant OR SNP OR polymorphism) AND (algeria or bahrain or egypt or iraq or jordan or kuwait or lebanon or or yemen or UAE or morocco or moroccan or oman or palestin or saudi or KSA or Syria or tunisia or sudan or djibouti or somali or mauritania or MENA or EMRO or arabs).
